# Prostate cancer evolution after COVID-19-related prostatitis in a TMPRSS2-altered patient: a case report and review of the molecular interface between SARS-CoV-2 and prostate oncogenesis

**DOI:** 10.3389/fonc.2025.1679663

**Published:** 2025-11-05

**Authors:** Fabricio Borges Carrerette, Magda Conceição Barbosa Gomes, Romulo Varella de Oliveira, Fabio Santiago, Janice Chicarino Coelho, Daniela Bouzas Rodeiro, Ana Beatriz da Silva Polonia, Felipe Vaz Chilão Guedes, Alexandre Rodrigues Oliveira

**Affiliations:** Urology Service, Pedro Ernesto University Hospital (HUPE), Department of Surgical Specialties, State University of Rio de Janeiro (UERJ), Rio de Janeiro, Brazil

**Keywords:** SARS-CoV-2, TMPRSS2, COVID-19, prostatitis, prostate cancer, ERG fusion, PTEN, novel hormonal agent

## Abstract

**Background:**

SARS-CoV-2 exploits TMPRSS2, an androgen-regulated protease highly expressed in prostate tissue, to enter host cells. While inflammation is a recognized promoter of oncogenesis, the possibility that viral prostatitis could precede prostate cancer has not been previously reported.

**Case presentation:**

We describe the case of a 55-year-old male with no family history of prostate or breast cancer and no germline pathogenic variants on next-generation sequencing (NGS), who developed lower urinary tract symptoms (LUTS) and PSA elevation shortly after a second COVID-19 infection. Multiparametric MRI initially demonstrated diffuse PI-RADS 4 changes compatible with prostatitis. Although symptoms improved with antibiotics, LUTS persisted and were managed with finasteride and doxazosin. Over the following two years, serial imaging revealed progression to a long, poorly demarcated PI-RADS 5 lesion extending from apex to base in the right posterior peripheral zone, and a smaller PI-RADS 4 lesion on the left. Targeted biopsy confirmed acinar adenocarcinoma (Gleason 7 and 6 in 16 of 26 cores). PET-PSMA showed disease confined to the prostate. The patient underwent neoadjuvant therapy with androgen deprivation therapy (ADT) plus a novel hormonal agent (NHA) from April 14 to October 15, 2024, resulting in significant tumor reduction. Radical prostatectomy on November 1, 2024 revealed a small residual acinar adenocarcinoma focus with perineural invasion, negative surgical margins, and molecular evidence of TMPRSS2::ERG gene fusion and PTEN loss.

**Conclusion:**

This is the first documented case suggesting a potential link between COVID-19-related prostatitis and subsequent prostate cancer in a TMPRSS2::ERG-altered patient without hereditary predisposition. Although causality cannot be established, the findings highlight a hypothesis-generating interface between viral infection, inflammation, and oncogenesis that warrants further study.

## Introduction

COVID-19, caused by SARS-CoV-2, is a multisystem disease in which viral entry depends on angiotensin-converting enzyme 2 (ACE2) and transmembrane protease serine 2 (TMPRSS2) (1). TMPRSS2 is of particular interest because it is androgen-regulated, highly expressed in the prostate, and a known driver of oncogenesis through gene fusions with ETS transcription factors, especially ERG (2, 3). The TMPRSS2::ERG fusion is one of the most frequent genomic alterations in prostate cancer and contributes to oncogenic transformation, particularly when combined with PTEN loss (4, 5).

Inflammation is a recognized factor in carcinogenesis, with chronic prostatitis implicated as a possible promoter of neoplastic transformation (4). The overlap between COVID-19-associated inflammation and oncogenic pathways in the prostate has therefore raised scientific concern (2, 3). Several studies have hypothesized that SARS-CoV-2 infection may influence prostate carcinogenesis through TMPRSS2 dysregulation and inflammatory cascades (1–3). Recent genomic reviews have also emphasized this potential mechanistic link (3).

To our knowledge, however, no previous case has been reported in which COVID-19-related prostatitis preceded the diagnosis of prostate cancer in a patient with a TMPRSS2::ERG fusion. We describe such a case, emphasizing the timeline of disease evolution, the absence of hereditary predisposition, and the hypothesis-generating implications for viral infection and oncogenesis.

## Case presentation

A 55-year-old male physician (FBC, also the first author), with no family history of prostate or breast cancer and no identified germline pathogenic variants on NGS of 421 cancer-related genes, experienced his first episode of COVID-19 in May 2020, presenting with systemic viral symptoms but no urinary complaints. His PSA was 2.5 ng/mL in August 2021.

**Figure 1:**
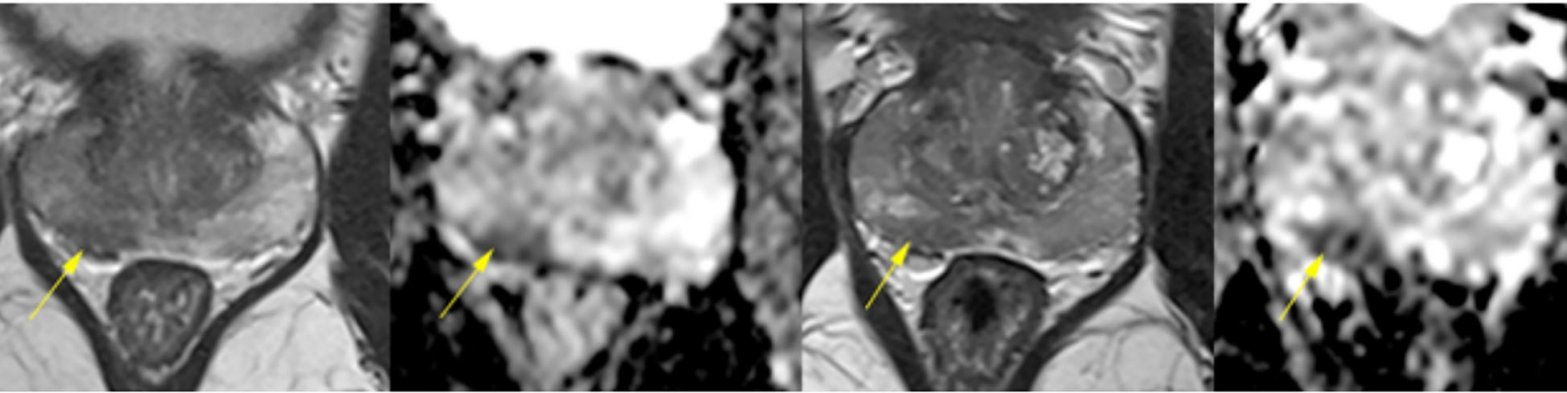
Multiparametric prostate MRI scans from June 22 and December 26, 2022, demonstrating diffuse and heterogeneous low signal intensity in the peripheral zone, predominantly on the right, with confluent, ill-defined elongated areas lacking discrete nodules. Both scans revealed diffuse early contrast enhancement and restricted diffusion at the right base (1.2 cm), consistent with PI-RADS 4 classification. The stability of these nonspecific findings over time, along with their bilateral distribution, suggested an underlying inflammatory or infectious process rather than malignancy.

In June 2022, during a second confirmed SARS-CoV-2 infection, he developed prostatitis with perineal pain, weak urinary stream, nocturia, and PSA elevation (peak 7.9 ng/mL in July 2022). Multiparametric MRI performed on June 22, 2022, and repeated on December 26, 2022, revealed diffuse PI-RADS 4 changes in the peripheral zone, predominantly on the right, interpreted as inflammatory or infectious in origin (**Figure 1**). Symptoms improved after antibiotic therapy, while persistent LUTS due to prostatic enlargement were managed with finasteride and doxazosin, which were continued until biopsy.

**Figure 2:**
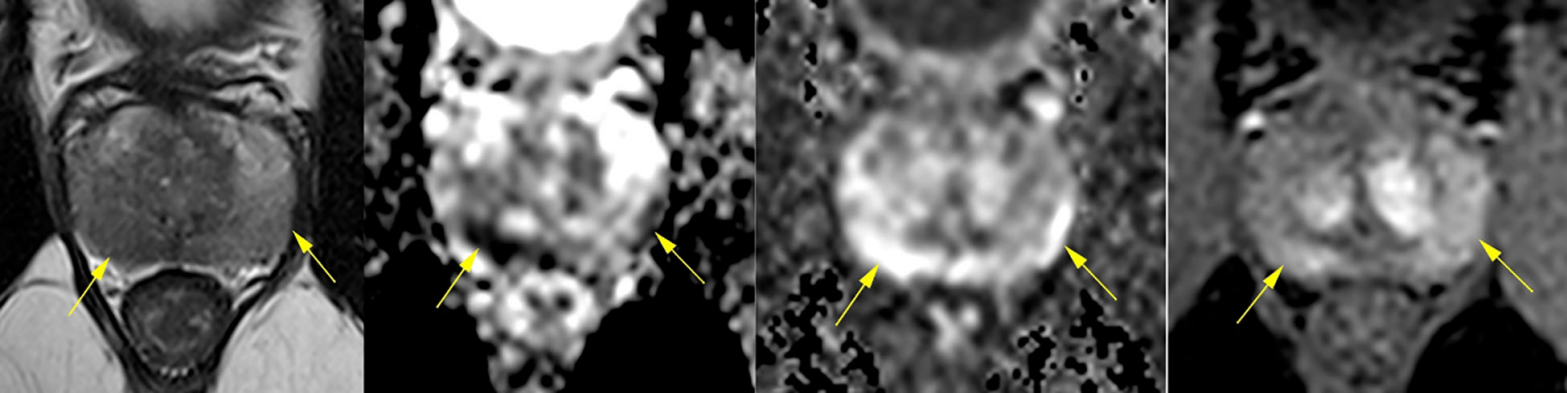
Multiparametric MRI scan from March 15, 2024, showing significant progression compared to prior studies. A long, poorly demarcated PI-RADS 5 lesion (2.2 × 0.8 × 1.7 cm) was identified in the right posterior peripheral zone, extending from apex to base, with capsular contact, low T2 signal, restricted diffusion (DWI/ADC), and early dynamic contrast enhancement (DCE). A smaller PI-RADS 4 lesion was also noted on the left, consistent with multifocal disease. These findings contrast with the diffuse inflammatory changes of **Figure 1** and support malignant transformation.

A third multiparametric MRI on March 15, 2024, demonstrated disease progression, with a long, poorly demarcated PI-RADS 5 lesion in the right posterior peripheral zone (2.2 × 0.8 × 1.7 cm), extending from apex to base, showing low T2 signal, restricted diffusion, and early contrast enhancement. A smaller PI-RADS 4 lesion was also identified on the left, consistent with multifocal disease (**Figure 2**). Prostate biopsy confirmed acinar adenocarcinoma (Gleason 7 and 6 in 16 of 26 cores). Staging with PET-PSMA (May 2024) showed disease confined to the prostate.

**Figure 3:**
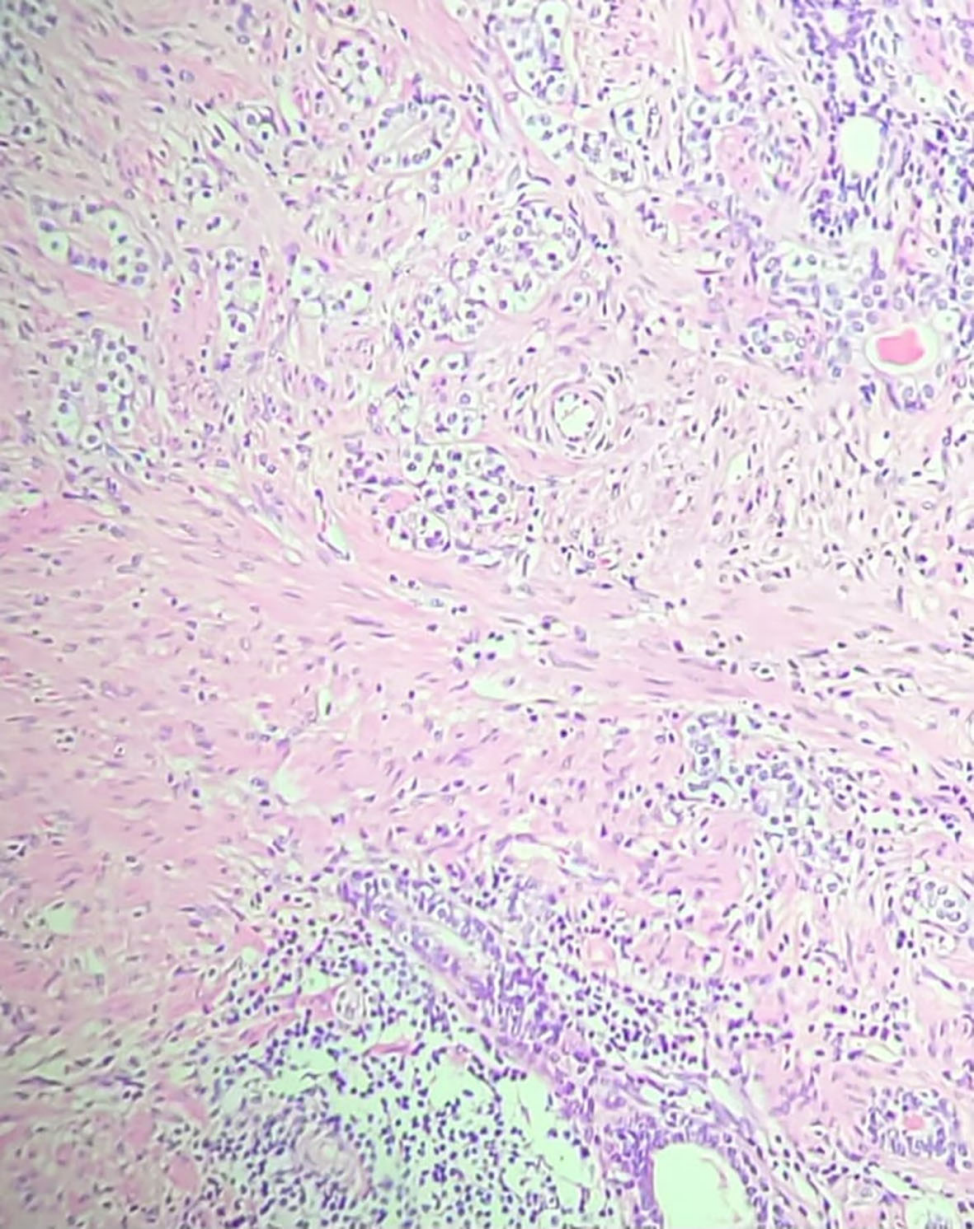
Hematoxylin and Eosin (H&E) stained section (20× magnification) from the radical prostatectomy specimen, demonstrating acinar adenocarcinoma with Gleason score 7 (3 + 4), ISUP grade group 2. Adjacent areas showed chronic inflammatory infiltrates consistent with prostatitis, supporting the hypothesis that COVID-19–related inflammation may have contributed to carcinogenesis in this patient.

The patient underwent neoadjuvant therapy with ADT plus a novel hormonal agent from April 14 to October 15, 2024, resulting in significant tumor shrinkage. He subsequently underwent radical prostatectomy on November 1, 2024. Surgical pathology revealed a residual 9 mm acinar adenocarcinoma focus with perineural invasion, but no vascular invasion. Surgical margins, seminal vesicles, and deferent ducts were negative. Background findings included basal cell hyperplasia, acinar atrophy, mild lymphohistiocytic inflammation, and stromal remodeling. Molecular testing confirmed a TMPRSS2::ERG fusion and PTEN loss (**Figure 3**).

A detailed chronological summary of clinical, imaging, and pathological findings is presented in **Table 1**.

**Table 1:** Clinical timeline of the case.

Date	Event/examination	Main findings/management
13/05/2020	First COVID-19 infection	Febrile viral illness, no urinary symptoms
17/08/2021	PSA	2.5 ng/mL
08/06/2022	Second COVID-19 infection + prostatitis	Perineal pain, LUTS, PSA 5.4 ng/mL
19/06/2022	PSA	6.0 ng/mL
22/06/2022	MRI (1st)	Diffuse PI-RADS 4 changes, prostatitis suspected
14/07/2022	PSA peak	7.9 ng/mL
Jul 2022	Treatment	Antibiotics; initiation of finasteride + doxazosin (continued until biopsy)
09/08/2022	PSA	6.7 ng/mL
06/09/2022	PSA	3.5 ng/mL
26/12/2022	MRI (2nd) + PSA	PI-RADS 4 changes; PSA 2.9 ng/mL
03/03/2023	PSA	2.1 ng/mL
15/03/2024	MRI (3rd) + PSA	Long, poorly demarcated PI-RADS 5 lesion (2.2 × 0.8 × 1.7 cm) + smaller PI-RADS 4; PSA 2.3 ng/mL
01/04/2024	Prostate biopsy	Adenocarcinoma Gleason 7 and 6, 16/26 cores positive
09/05/2024	PET-PSMA	Uptake confined to prostate, no metastases
14/04–15/10/2024	Neoadjuvant therapy	ADT + novel hormonal agent → significant tumor volume reduction
01/11/2024	Radical prostatectomy	Residual 9 mm acinar adenocarcinoma, perineural invasion, negative margins; TMPRSS2::ERG fusion, PTEN loss

## Discussion

This case illustrates a unique sequence in which COVID-19-related prostatitis was followed by the diagnosis of prostate cancer in a patient with a TMPRSS2::ERG gene fusion. While causality cannot be established from a single report, the chronological association is noteworthy and highlights the potential intersection of viral infection, chronic inflammation, and oncogenesis.

TMPRSS2 facilitates SARS-CoV-2 entry into host cells (1) and is simultaneously one of the most frequent genomic drivers in prostate cancer through its fusion with ERG (2, 3). This dual role positions TMPRSS2 as a potential molecular bridge linking infection and oncogenesis (1–3, 5).

Inflammation is a well-recognized promoter of carcinogenesis. In the prostate, chronic prostatitis has been hypothesized to trigger neoplastic transformation (4, 5). Our patient developed significant prostatitis temporally associated with COVID-19 infection, with imaging and PSA fluctuations consistent with inflammatory injury (4). Notably, the surgical specimen demonstrated PTEN loss, a common co-driver of aggressive disease when combined with TMPRSS2::ERG fusion, supporting the concept of multi-hit oncogenesis (1–3).

Neoadjuvant therapy with ADT plus a novel hormonal agent from April to October 2024 led to significant tumor shrinkage, with only a small residual focus of adenocarcinoma identified at surgery. This highlights the responsiveness of intensified androgen blockade in this molecular context. The absence of extraprostatic extension or positive margins indicates a favorable pathological outcome.

This report has important limitations. First, it represents a single case, which is insufficient to establish causality. Second, the patient had no prior prostate biopsies or imaging before COVID-19, making it impossible to exclude pre-existing subclinical cancer. Third, there was no comparison with patients who developed prostate cancer after non-COVID-related prostatitis, or with healthy controls. Importantly, the patient had no family history of prostate or breast cancer and no germline variants detected on NGS, further supporting the possibility that viral prostatitis and inflammatory mechanisms, rather than hereditary predisposition, may have contributed to oncogenesis in this case. Despite these limitations, the case is strengthened by its comprehensive longitudinal documentation (PSA, imaging, biopsy, PET-PSMA, neoadjuvant therapy, surgery, and molecular analysis).

## Conclusion

This is the first reported case suggesting a potential association between COVID-19-related prostatitis, TMPRSS2::ERG fusion, and prostate cancer development in the absence of hereditary predisposition. While causality cannot be established, the temporal relationship highlights a hypothesis-generating link between viral infection, inflammation, and oncogenesis. Further studies are warranted to explore the molecular interplay between SARS-CoV-2 and prostate cancer.

## Data Availability

The original contributions presented in the study are included in the article/supplementary material. Further inquiries can be directed to the corresponding author.

## References

[1] Cheng J Zhou J Fu S Fu J Zhou B Chen H . Prostate adenocarcinoma and COVID-19: The possible impacts of TMPRSS2 expressions in susceptibility to SARS-CoV-2. J Cell Mol Med. (2021) 25:4157–65. doi: 10.1111/jcmm.16385, PMID: 33609069 PMC8013364

[2] Afshari A Janfeshan S Yaghobi R Roozbeh J Azarpira N . COVID-19 pathogenesis in prostatic cancer and TMPRSS2-ERG regulatory genetic pathway. Infect Genet Evol. (2021) 88:104669. doi: 10.1016/j.meegid.2020.104669, PMID: 33301988 PMC7720011

[3] Lee S Kim SY . Potential molecular links between SARS-CoV-2 infection and prostate cancer progression. Genomics Inform. (2022) 20:e30. doi: 10.5808/gi.22012, PMID: 36239107

[4] Daneshwar D Lee Y Nordin A . COVID-19 and prostatitis: A review of current evidence. Diseases. (2024) 12:157. doi: 10.3390/diseases12070157, PMID: 39057128 PMC11276594

[5] Chakravarty D Nair SS Hammouda N Ratnani P Gharib Y Wagaskar V . Sex differences in SARS-CoV-2 infection rates and the potential link to prostate cancer. Commun Biol. (2020) 3:374. doi: 10.1038/s42003-020-1088-9, PMID: 32641750 PMC7343823

